# Medication Reconciliation at Discharge from Hospital: A Systematic Review of the Quantitative Literature

**DOI:** 10.3390/pharmacy3020053

**Published:** 2015-06-23

**Authors:** Maja H. Michaelsen, Paul McCague, Colin P. Bradley, Laura J. Sahm

**Affiliations:** 1 Pharmaceutical Care Research Group, School of Pharmacy, University College Cork, College Road, Cork, Ireland; E-Mails: m.hoetoft@gmail.com (M.H.M.) ; pmccague01@qub.ac.uk (P.M.); 2 Department of Drug Design and Pharmacology Faculty of Health and Medical Sciences, University of Copenhagen, Universitetsparken 2, DK-2100 Copenhagen, Denmark; 3 School of Pharmacy, Queen’s University Belfast, Lisburn Road, Belfast, UK; 4 Department of General Practice, Department of General Practice,1st Floor, Brookfield Health Sciences Building, University College Cork, Cork, Ireland; C.Bradley@ucc.ie; 5 Department of Pharmacy, Mercy University Hospital, Grenville Place, Cork, Ireland

**Keywords:** Medication reconciliation, medication errors, medication discrepancies, review

## Abstract

Medicines reconciliation is a way to identify and act on discrepancies in patients’ medical histories and it is found to play a key role in patient safety. This review focuses on discrepancies and medical errors that occurred at point of discharge from hospital. Studies were identified through the following electronic databases: PubMed, Sciences Direct, EMBASE, Google Scholar, Cochrane Reviews and CINAHL. Each of the six databases was screened from inception to end of January 2014. To determine eligibility of the studies; the title, abstract and full manuscript were screened to find 15 articles that meet the inclusion criteria. The median number of discrepancies across the articles was found to be 60%. In average patient had between 1.2–5.3 discrepancies when leaving the hospital. More studies also found a relation between the numbers of drugs a patient was on and the number of discrepancies. The variation in the number of discrepancies found in the 15 studies could be due to the fact that some studies excluded patient taking more than 5 drugs at admission. Medication reconciliation would be a way to avoid the high number of discrepancies that was found in this literature review and thereby increase patient safety.

## 1. Introduction

Ensuring patient safety in the healthcare system is a complex challenge and involves many health care professionals across multiple institutions. One of the most common ways to prevent, manage and cure diseases and illnesses is by the use of medicines. This, however, does not come without its own set of risks. Medication errors (ME) are prevalent and costly; both in terms of patient injury and economics. Over half of these errors occur during transitions of care or at the interface of care [1,2,3,4]. According to Ferner and Aronson (2006), a medication error can be defined as “a failure in the treatment process that leads to, or has the potential to lead to, harm to the patient” [5]. This is in contrast to an adverse drug event (ADE), which is, “an actual unintended medication related event that occurs during treatment of the patient with pharmaceuticals”. The difference therefore lies in the event having taken place (ADE) rather than the error (which may result in an event) being noted (ME). An example of a medication error is when a patient has not been prescribed one of their regular medicines on admission to hospital; this would be an error of omission and an ME. However, if the patient had been prescribed a medicine (even at the correct dose) that resulted in the patient experiencing an unintended side-effect (which could be anything from pruritus to anaphylaxis), this would be classed as an adverse drug event (ADE) [6,7]. It has been documented that there is a positive association between the number of medicines prescribed for a patient and the likelihood of experiencing an ME [8]. A structured handover between carers is one way in which MEs can be reduced [9]. Medication reconciliation has been acknowledged as one of the key elements to improve patient safety by decreasing MEs at discharge and transitions of care [1,10].

Medication reconciliation is defined as the process of “creating the most accurate list possible of all medications a patient is taking—including drug name, dosage, frequency and route—and comparing that list against the physician’s admission, transfer and/or discharge orders, with the goal of providing correct medication to the patient at all transitions points within the hospital” [11]. Medication reconciliation consists of four steps that help to ensure patient safety across the healthcare system: (1) verification: the current medication list is obtained; (2) clarification: the medication and dosages are checked for adequacy; (3) reconciliation: newly-prescribed and previous medications are compared and documented; and (4) transmission: an updated and verified medication list is communicated to the next healthcare provider [11,12].

Two other terms that are commonly used when discussing medication reconciliation are: intentional and unintentional discrepancies. An unintentional discrepancy is one that occurs without the professional involved making a conscious decision to alter therapy. In contrast, those discrepancies that are made due to an underlying co-morbidity or change in drug metabolism (for example, a decline in renal function) are termed intentional. These intentional discrepancies will have been usually documented in a standard manner, in the medical notes, by the physician amending the prescription, as they are consciously making the decision to alter the therapy. Whilst only the unintended discrepancies can be classified as MEs, it is important to note that an intentional discrepancy may still lead to confusion and errors at discharge, if not documented clearly [7].

The aims of this literature review are to compile and analyse journal articles that focus on medication reconciliation at discharge. The objectives are to report the prevalence of ME and the mean number of discrepancies at discharge (expressed as the percentage of patients where available), as well as the most common types of errors and medications (or classes of medications) most frequently implicated.

## 2. Experimental Section

The following databases were used: PubMed, Sciences Direct, Excerpta Medica dataBASE (EMBASE), Google Scholar, Cochrane and, Cumulative Index to Nursing and Allied Health Literature (CINAHL). To ensure that all relevant articles were found, an advanced search strategy was undertaken. The search strategy is described in Table 1.

Articles had to meet the following inclusion/exclusion criteria. 

Inclusion criteria:

- Published in English
- Quantitative studies, *i.e.*, studies that examine the prevalence of ME, or the mean number of discrepancies at discharge (expressed as the percentage of patients where available), or studies that quantify the most common types of errors and medications (or classes of medications) most frequently implicated in these errors.

Exclusion criteria:

- Duplicates
- Articles not published in English
- Review articles, meta-analysis and editorials
- Qualitative studies

The titles and abstracts of the articles found in the searches were screened for relevance before final inclusion.

**Table 1:** Results of the search strategy, including: search terms, limits, results, relevant studies and included studies.

Database	Search Terms	Limits	Results	Relevant Studies	Included Studies
PubMed	Medication reconciliation AND discharge AND error *	English	105	12 (8 articles were duplicates from other databases)	4
Science Direct	Medication reconciliation AND discharge AND error *	Search in author, title and keywords	34	3	3
Cochrane	Medication reconciliation AND discharge AND error *	Search in author, title and keywords	14	2 (both duplicates from other databases)	0
Cumulative Index to Nursing and Allied Health Literature(CINAHL)	Medication reconciliation AND discharge AND error *	English	25	5	5
Excerpta Medica dataBASE (EMBASE)	Medication reconciliation AND discharge AND error *	Advanced search English	51	10 (all duplicates from other databases)	0
Google Scholar	With all words: error * With the exact phrase: medication reconciliation Where my words occur: in the title of the article		35	4 (1 was a duplicate from another database)	3
Total = 15					

The asterisk (*) is used to allow for terms that have error or errors.

**Figure 1:**
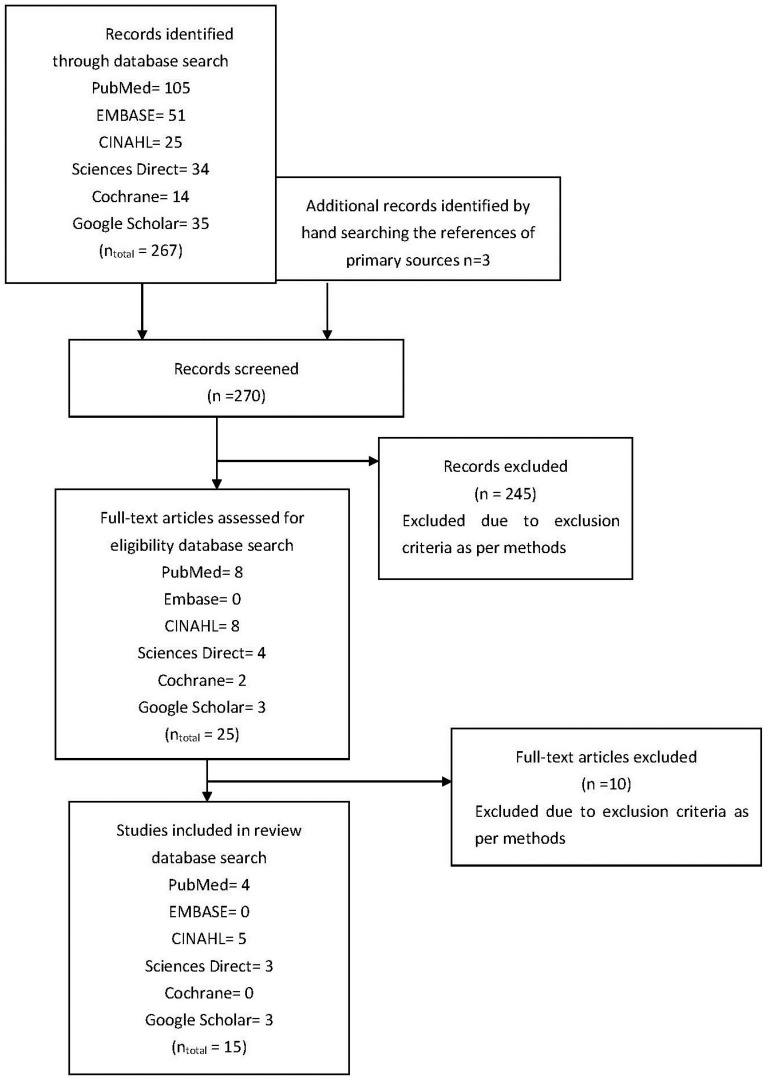
Flow diagram of studies assessed and included.

Figure 1 shows a flow chart of the selection of relevant articles.

## 3. Results and Discussion

The key results from each article included in the literature review are summarised in Table 2.

This literature review was conducted to give an overview of the quantitative research on medicine reconciliation at hospital discharge. During the review, 270 articles were screened, after the advanced search strategy was conducted; see Figure 1. This resulted in the inclusion of 15 articles for this review (Table 2). The data from these 15 articles represent international data from over 6000 hospital discharges.

**Table 2:** Presentation of results. Key elements for each article are described.

Year	Study	Study Design	Study Population
Climente-Martí *et al.*, 2010 [13].	Potential Risk of Medication Discrepancies and Reconciliation Errors at Admission and Discharge from an Inpatient Medical Service [13]	Observational prospective study Preadmission treatment was compared with treatment prescribed on admission and discharge	Country: Spain 120 patients included Mean age: 76 years (SD 14.4) Mean number of preadmission drugs: 7.5
**Key findings:** 46 patients had a discrepancy at discharge corresponding to 38.3%. The most common therapeutic groups related to reconciliation errors were: blood/hematopoietic organ drugs (30%), cardiovascular (20%) and gastrointestinal agents (20%). **Limitations:** Medication list prior to admission was done through an interview with the patient or care giver; they might not always be able to give the correct information. Small sample size (*n* = 120) meant lack of power in the study. Clinical significance of the errors was decided in a group, which could lead to subjective bias.			
Cornu *et al.*, 2012 [14].	Effect of Medication Reconciliation at Hospital Admission on Medication Discrepancies During Hospitalization and at Discharge for Geriatric Patients [14]	Retrospective single-centre cohort study Medication reconciliation at admission, during hospitalization and at discharge was conducted by an independent pharmacist	Country: Belgium 199 patients were included Mean age: 83.7 years (SD 5.8) Mean number of preadmission drugs: 7.2
**Key findings:** 278 of the 682 discrepancies at admission resulted in discrepancies at discharge (40.8%). The reconciliation process at discharge revealed 554 discrepancies and a mean of 3 per patient. Omitted drugs were the cause in 47.7% of the errors at discharge. All types of discrepancy except for “drugs prescribed even though discontinued” occurred more often at discharge than during hospitalization. For every additional drug in the medication history, the likelihood of a discrepancy increased by 47%, but for every additional source used to make the medication history, the likelihood of a discrepancy decreases by 78%. **Limitations:** Recall bias; the doctor made a medication history prior to the pharmacist.			
Vira *et al.*, 2006 [15].	Reconcilable Differences: Correcting Medication Errors at Hospital Admission and Discharge [15]	Prospective study At discharge, pre-admission and in-patient medications were compared with discharge orders and written instruction	Country: Canada 60 patients included in the study only 56 at discharge Mean age: 56 years (SD 24)
**Key findings:** 41% had at least one unintended discrepancy at discharge, and the mean per patient was found to be 1.2. Types of unintended discrepancies: omitted medication/prescription (45%), lack of discharge instructions (51%) and incorrect details of frequency/dose/route (4%). **Limitations:** Clinical importance of the interventions was judged by a single review. Results are not generalizable due to the small sample size.			
Kripalani *et al.*, 2012 [16].	Effect of a Pharmacist Intervention on Clinically Important Medication Errors after Hospital Discharge [16]	Randomized, controlled trial Pharmacist-assisted medication reconciliation	Country: United States of America (USA) 851 patients included Mean age: 60 years (SD 14.4)
**Key findings:** Between 0.87 and 0.95 clinically-important errors were found per patient; 50.8% had at least one clinically-import error after discharge. 30.3% of the patients experienced a preventable adverse drug event after discharge; 13% of these resulted in a visit to the emergency department. 424 (29.7%) potential adverse drug events (ADEs) were found, half of which were related to medication discrepancies and half of which were related to non-adherence. The most common medication discrepancies were found to be: omission (34.5%), incorrect dose (32.9%), incorrect frequency (15.9%) and additional medicine that should not be on the list (11.9%). **Limitations:** The patient population had acute cardiovascular problems. The types of interventions may differ from other wards and make generalization difficult. Not all patients received the full intervention as intended			
Karapinar-Carkit *et al.*, 2009 [10].	Effect of Medication Reconciliation with and without Patient Counselling on the Number of Pharmaceutical Interventions among Patients Discharged from the Hospital [10]	Prospective observational study To examine the effect of medication reconciliation with and without patient counselling	Country: Netherlands Including 262 patients Mean age: 65 years (SD 17.3) Mean number of drugs preadmission: 6.6 Mean number of drugs at discharge: 9.1
**Key findings:** For patients without counselling, the number of intervention after medication reconciliation at discharge was 2.7 per patient, compared to 5.3 interventions per patient whom received counselling. 72.5% of the study population had discrepancies at discharge; the most common were that the physician forgot to restart medication, which had been temporarily discontinued during hospitalization. Interventions for stopping a drug were made for 55% of the patients with patient counselling and 41.6% for the patients without; the most common drugs stopped were: laxatives (13.5%), gastric acid suppressants (13.1%) and sedatives (7.6%); the reason for stopping these drugs was “no indication”. **Limitations:** The study did not assess the impact on patient outcome after intervention. High exclusion rate, *i.e.*, patients living in nursing homes were not included in this study.			
Pippins *et al.*, 2008 [17].	Classifying and Predicting Errors of Inpatient Medication Reconciliation [17]	Prospective observational study To compare medication histories with admission and discharge orders	Country: USA 180 patients were included <50 years: 21% 50–60 years: 20% 60–75 years: 22% >75 years: 37% Number of discharge drugs: 11
**Key findings:** 2066 medication discrepancies were detected; 45% of these were classified as unintentional and 27% of these had potential harm for the patient. 54% of the patients had at least one potential adverse drug event (PADE). Most PADEs were due to errors in taking the preadmission medication history (72%). PADEs divided in to therapeutic categories: cardiovascular (20%), respiratory (9%), gastrointestinal (8%), lipid-lowering (6%) and antidepressant (5%) **Limitations:** Patients with a short stay at the ward were not included; this could lead to a study population with more complex medication and diseases. Study measured PADEs and not actual ADEs.			
Salanitro *et al.*, 2012 [18].	Effect of Patient- and Medication-Related Factors on Inpatient Medication Reconciliation Errors [18]	Cross-sectional analysis To identify medication-related factors that contribute to pre-admission medication list error and to test whether this error exists in the discharge medication list	Country: USA 423 patients were included Mean age: 61 years (SD 14) Mean number of preadmission drugs: 8 Mean number of discharge drugs: 10
**Key findings:** 158 patients (40%) had a medication error (ME) at discharge. The number of preadmission list errors and the number of medication changed during the hospitalization were significantly associated with the number of MEs identified at discharge. The number of MEs at discharge was less common for people living alone and people with cognitive impairment .**Limitations:** The pharmacist classified the severity of clinical relevant MEs, which could lead to subjective bias. This study was conducted at a hospital with electronic medical records and may differ from hospitals with a paper-based medical record.			
Paiboonvong *et al.*, 2009 [19].	Incidence of Medication Errors in Medication Reconciliation at General Medical Wards, Ramathibodi Hospital [19]	Prospective descriptive study To determine MEs through medication reconciliation during transition phases	Country: Thailand 107 patients were enrolled in this study Mean age: 57.3 years (SD 19.6) Mean number of pre-admission drugs: 6.2
**Key findings:** Intentional discrepancies were found in 69.5%, and unintentional discrepancies were found in 89.5% of the patients. In 16% of the patients, an ME was identified at discharge with a mean number of MEs per patient of 1.1; the class of drug most often causing MEs was anti-hyperglycaemic drugs (33.3%). Types of MEs identified at discharge: omission (55.6%) and different route, dose or frequency (44.4%) 94.4% of the MEs were prevented by the pharmacist. **Limitations:** A small study population; bias in the inclusion of patients. No interviews with patients to confirm the medication list at admission.			
Stitt *et al.*, 2011 [20].	Medication Discrepancies Identified at Time of Hospital Discharge in Geriatric Population [20]	Retrospective review of a random cohort Identify discharge medication list discrepancies	Country: USA A randomized population of 200 patients was included Mean age: 77.2 years (range 66–97) Receiving on average 13.4 medications
**Key findings:** In all, 1923 medication discrepancies were reported in the discharge summary, discharge orders and medication list, during the study; the most common was in relation to the route of administration. 1380 of the discrepancies were found in the physician discharge summary, whereas only 191 were found in the physician discharge orders. A linear relationship was found between the number of medications at time of discharge and the number of medication discrepancies (*p*-value = 0.001) 55 patients were in wards with a pharmacist, and a significantly lower number of discrepancies per patient was found for these patients. Medication discrepancies divided into therapeutic categories: central nervous system (21.1%), cardiovascular (20.8%), nutrients (13.5%), endocrine/metabolic (11.5%), gastrointestinal (8.5%), respiratory (8.4%), haematological (5.2%), renal (4.9%), anti-microbial (3.6%) and others (2.5%) **Limitations:** Retrospective design, which made it impossible to detect if a pharmacist was present for all patients during discharge at the “pharmacist-ward”. During the discharge preparation time, final medication changes may occur after the medicine list has been printed, and this could lead to a change in the number of discrepancies.			
Knez *et al.*, 2011 [7].	The Need for Medication Reconciliation: A Cross-Sectional Observational Study in Adult Patients [7]	Prospective descriptive cross-sectional observational study Pre-admission therapy was compared with in-patient and discharge therapy	Country: Slovenia 101 patients were included Median age: 73 years (IQR: 65–79) Median number of pre admission drugs: 6
**Key findings:** Overall, the study population was prescribed 747 drugs at discharge, and it was found that 75.8% (*n* = 566) was in discordance with pre-admission therapy. At discharge, the percentage of MEs was 65.2%, and of this, 58.0% was rated to be clinically important. 84.2% of the patients were found to have one ME, and a median of 3 MEs per patient was recorded. Reasons for discrepancies at discharge were found to be: drug omission (28.8%), discrepancy in dose (13.8%), drug substitution (14.7%) and others (15.4%). Reasons for MEs: drug omission (40.4%), discrepancy in drug dose (15.7%) drug omission (17.9%), drug substitution (13.3%) and others (12.7%). **Limitations:** Only 101 patients were included, and it was only performed at a single site.			
Herrero-Herrero *et al.*, 2010 [21].	Medication Discrepancies at Discharge from an Internal Medicine Service [21]	Descriptive retrospective study Review on non-selected discharge reports to find discrepancies	Country: Spain Data from 790 patients corresponding to 954 discharge reports were included. Median age: 83 (SD 11) Median number of pre admission drugs: 6 Median number of discharge drugs: 7
**Key findings:** In 832 cases (87.2%), discrepancies were found in the reconciliation process (including justified and unjustified discrepancies). Intentional discrepancies were recorded in 828 (median 3; range 0–13 discrepancies per discharge report). Unintentional discrepancies were recorded in 52 (5.4%) discharge reports. The most frequent medication reconciliation error was drug omission (84.6%). The 5.4% with unintentional discrepancies was found to have a significantly higher number of permanent medication at admission, 7.5 medications against 6.2 (*p*-value = 0.005) **Limitations:** Chronic medication was not looked at when doing the medication reconciliation. This could be an explanation for the low number of errors recorded. This hospital may not be representative of all hospitals, because medication reconciliation is already an incorporated part of the work day.			
Allende Bandrés *et al.*, 2013 [1].	Pharmacist-Led Medication Reconciliation to Reduce Discrepancies in Transitions of Care in Spain [1]	Retrospective descriptive study The object was to quantify and analyse reconciliation unjustified discrepancies	Country: Spain Reviewed 1150 patients treatment Average age: 76.4 years (range 15–101)
**Key findings:** 1 in 5 patients had a discrepancy, and the reconciliation errors per patient w 1.93. Most frequently unjustified discrepancies (reconciliation errors): incomplete prescriptions (63.86%), omission (16.63%), different dosage/frequency/route (10.51%), wrong drug (1.85%) and other (0.35%). Therapeutic groups most often associated with medication reconciliation interventions: cardiovascular system (CVS) (34.02%) and CNS (21.85%). **Limitations:** Only unjustified discrepancies were recorded, and this could lead to an underestimate compared to other studies. Medication reconciliation at discharge was only conducted for patients taking more than 5 drugs (*n* = 1150); at admission, medication reconciliation was conducted for all included patients (*n* = 2573).			
Bjeldbak-Olesen *et al.*, 2013 [22].	Medication Reconciliation Is a Prerequisite for Obtaining a Valid Medication Review [22]	Retrospective study Comparing medication review with medication reconciliation	Country: Denmark 75 patients included in the study Mean age: 71.7 years Mean number of pre admission drugs: 5.9
**Key findings:** 198 discrepancies were identified during the study; 2.6 discrepancies per patient. 109 undocumented changes were found (changes where the physician did not state any reason). 86.7% of the patients had a discrepancy, and 69.3% had between 1 and 6. Types of discrepancies: omission of drug in discharge summery, extra/analogous drug in discharge summary, non-recognizable drug, uncertainty of dosage, unnecessary drug and other. **Limitations:** Categories of errors are empirically based. Small study population; a bigger study population would give the study more power and validity.			
Geurts *et al.*, 2013 [23].	Medication Reconciliation to Solve Discrepancies in Discharge Documents after Discharge from the Hospital [23]	Retrospective single-site study To evaluate the number, type and origin of discrepancies in discharge documents	Country: Netherlands 100 discharge documents (83 patients) Mean age: 63.1 years (SD 17) Mean number of pre admission drugs: 7 Mean number of discharge drugs: 8.3
**Key findings:** 223 discrepancies were detected, correlating to 2.2 discrepancies per discharge. 155 out of 223 (69.5%) led to a change in the discharge medicine after the pharmacy had contacted the patients’ medical specialist. For each additional drug added after discharge, there was an increase of 9.8% in the number of interventions preformed. **Limitations:** Small study population. Single site study.			
Grimes *et al.*, 2011 [24]	Medication Details Documented on Hospital Discharge: Cross-Sectional Observational Study of Factors Associated with Medication Non-Reconciliation [24]	Cross-sectional observational healthcare record review survey Medication non-reconciliation, prescribing errors at discharge or lack of document for changes	Country: Ireland 1246 episodes of care were investigated Median age: 62 years (Range 16–96) Median number of pre admission drugs: 5
**Key findings:** In 624 (50.1%) of the cases examined, a minimum of one medication was found; the most common reason was omission of a drug at discharge. Patients with a chronic condition were twice as likely to experience problems with reconciliation as patients with an acute condition. For each additional medication, a patient had a 26% increased likelihood of experiencing non-reconciliation. **Limitations:** The Hawthorne effect could lead to an underestimation of non-reconciliation.			

Of the included studies, six were retrospective [1,14,20,21,22,23], and six were prospective studies [7,10,13,15,17,19]. The advantages of these types of studies include the ability to examine multiple outcomes of a single risk factor. The disadvantages of retrospective studies include: (i) if records that were not designed for the study are used, the available data may be of poor quality; (ii) there can be frequently an absence of data on potential confounding factors; and (iii) it may be difficult to identify an appropriate exposed cohort and an appropriate comparison group. Disadvantages of prospective cohort studies include: (i) having to follow large numbers of subjects for a long time; (ii) expensive and time consuming; (iii) not good for rare diseases or diseases with a long latency; and (iv) differential loss to follow up can introduce bias.

Furthermore, one study was a randomised clinical trial (RCT) [16], and two were cross-sectional observational studies [18,24]. An RCT is classified as the gold standard in the clinical setting, as it can greatly reduce the confounding factors by random assignment and, hence, make the study and control groups equivalent at study outset. This (plus any additional blinding) enables a cause and effect relationship to be established. However, the challenge with RCTs in medication reconciliation (MR) studies is that, in effect, you have to conduct MR (or a form thereof) on the controlled group to elicit medication-related problems, and so, most studies are not described as RCTs. Observational studies however reach a conclusion by comparing subjects against a control group, in cases where the researcher has no control over the experiment. One of the primary drivers for undertaking observational research is ethical concerns. One of the main challenges associated with observational studies is that the investigator has no control over the configuration of the control groups and cannot randomise the allocation of subjects. This can lead to bias and may also mask cause and effect relationships or, alternatively, suggest associations where there are none.

The number of included patients/discharges in each article varied from 60–1246. The reason for the big differences in numbers of included subjects can be explained by the fact that some of the studies were conducted at a single site and for a short period of data collection, as evidenced in the work of Vira *et al.* [15]. In contrast, the work presented by Grimes *et al.* [24] was conducted at two sites over a 29-month period. Another key difference is that in some articles, they have looked at patients, whereas other articles have looked at discharges during the study period. This means that one patient could appear in the study on more than one occasion. It is well established that increasing the sample size/study population increases the power of the study and the ability to detect with any degree of accuracy the differences between sample cohorts [25]. The mean age of the study populations for the fifteen articles varies from 56 years (standard deviation (SD) 24 years) [15] to 84 years (SD 5.8 years) in the Cornu *et al.* (2012) [14] study. The mean age in the studies included in this review is 69 (SD 9.1) years, which indicates that overall, the study population would be categorized as elderly. Due to the fact that patients taking five or more drugs are increasingly likely to be found in the older population, it is not surprising that the number of preadmission drugs are high and varies from five [24] to eight drugs per patient [18].

One of the factors identified as having a significant impact on the risk of discrepancies and ME at discharge is the number of drugs a patient is prescribed. For every additional drug, a patient is taking the risk of being exposed to discrepancies, which could lead to increased ME. Cornu *et al.* has identified this increased risk to be 47% for each additional drug prescribed [14]. Similarly, Stitt *et al.* refer to a linear relationship between the number of drugs prescribed at discharge and the volume of discrepancies at discharge [20]. A Canadian study has shown an increase in the likelihood of potential drug–drug interactions for patients aged more than 50 years due to the significant increase in the number of medication used per patient [26]. Whilst this finding is not a surprise and follows a simple proportionality association, e.g., the more drugs prescribed, the more drugs errors identified, it is worth noting that this is the case.

Ideally, the same variables would have been collected within each of the 15 studies, and the studies would have been conducted in the same way. This would ensure that a direct comparison would be possible. However, this is not the case, as there are methodological variances between the studies, which, in turn, leads to the variances in the data extracted from the each study. Specifically, the use of non-standard MR processes in Climente-Martí *et al.* (2010) [13], Cornu *et al.* (2012) [14], Paiboonvong *et al.* (2009) [19] and Herrero-Herrero *et al.* (2010) [21] is a further limitation.

One of the crucial differences between the studies is the way in which discrepancies were designated to be “intentional” or “unintentional”. In most studies, a panel including physicians and pharmacists decided if a discrepancy qualified as intentional or unintentional. This allowed for a range of healthcare professionals to be involved and, hence, is more likely to imitate usual care and take into account the views and knowledge of a range of stakeholders. In Herrero-Herrero *et al.* [21], the treating physician was solely in charge of performing the medication reconciliation, which could lead to a potential bias in the error-reporting mechanism. These differences in the classification systems and the level of consensus involved in the reporting may account for the variation of the results in between studies. To conduct a research study on medication, reconciliation guidelines regarding inclusion and exclusion of patients have to be structured. Ideally, these criteria are made to ensure that the study population reflects the population that is of interest. In the study conducted by Pippins *et al*., patients with “short stays” at the ward were not included; the definition of a short stay was not specified in the article, but this could lead to an overestimate of discrepancies, because only those patients who were relatively more complex (and hence, had a longer stay) were included [17]. On the other hand, the exclusion of patients from nursing homes and patients taking more than five drugs could potentially lead to a significantly lower number of both MEs and discrepancies than would otherwise be the case [1,10,27].

For the six prospective studies included in this review, it is important to consider the Hawthorne effect, as suggested by Grimes *et al.* [24]. This effect can result in modified behaviour of those under observation and, hence, may distort the findings; potentially leading to an underestimate of the errors found.

By comparison of the preadmission medication list against the discharge medication list, discrepancies are identified. After a discrepancy is identified, it might lead to an ME, but this is not always the case. For example, new medications appearing on the discharge list (compared to the admissions list) would be categorised as an intentional discrepancy, but not an ME. Focusing on unintentional discrepancies only, the results of Allende Bandrés *et al.* [1] (20%) could have been unrealistically low due to the exclusion of patients taking more than five drugs at discharge and the recording of unjustified/undocumented discrepancies only. In the same way, Bjeldbak-Olsen *et al.* [22] may have overestimated the number of discrepancies by including both intentional and unintentional discrepancies, thereby reporting 86.7% discrepancies at discharge, which is higher than most studies. As the range of discrepancies varies widely and is not uniformly reported, the median value (60%) is possibly the most useful.

The variation in the inclusion and exclusion criteria of the studies examined further contributes to the wide range of reported discrepancies at discharge. If only simple (*versus* multi-morbid) patients treated with fewer than five medications are included, the number of discrepancies was lower [14]. The average number of discrepancies detected per patient varies from 1.2–5.3, although five of the seven studies that measured average discrepancy per patient found it to be around 2–3. This implies that the majority of patients who leave hospital may have more than one medication discrepancy and, therefore, could be at risk of an ME. This is supported by other studies that state that the likelihood of an ME or potential adverse drug events (PADE) increases for patients with medication discrepancies [17,28]. In addition, the variation in the geographical location of the studies under consideration may also contribute to the differences observed.

The most common discrepancy described in the literature is omission of medication. Omission was found to be the reason for discrepancies in up to 56% of the cases in one study [19]. The other major reasons for discrepancies were inaccuracies related to route/frequency/dose of medicines; one study found that these three factors were the reason for up to 44% of the discrepancies [19]. These findings are supported by other studies, where the main reasons identified for discrepancies in medical history were identified as omitted medication, as well as inconsistencies in route/frequency/dose [29,30].

The therapeutic group most frequently associated with errors regarding medication reconciliation are cardiovascular system (CVS) drugs [1]. The frequency of errors due to CVS drugs was between 20% and 34% [1,13]. The prevalence of cardiovascular diseases (CVD) worldwide is increasing, and some even describe CVD as an epidemic [31]. Many CVD patients are treated with multiple drugs, which increases the risk of ME [32]. Other therapeutic classes involved in errors during medication reconciliation are central nervous system (CNS) drugs (21%) [20] and gastrointestinal (GI) drugs (8%–20%) [10,14]. An Irish study was conducted based on the Health Service Executive (HSE)-Primary care reimbursement services (PCRS) pharmacy database. HSE-PCRS provides free or reduced health services to an estimated 1.2 million people in the Republic of Ireland (accounting for approximately 26% of the total population). This study found that the four most common therapeutic areas that patients were treated for were CVS, CNS, musculoskeletal and GI. Furthermore, this study estimated that three-quarters of the elderly population (defined in this dataset as those aged greater than 70 years) suffer from two or more chronic diseases [33]. The Institute for Safer Medication Practices (ISMP), a non-profit organization in the United States of America working for increased patient safety, has made a list of high-risk drugs according to the Anatomical Therapeutic Chemical Classification system (ATC). When looking at this list, more than 50% of the drugs on the list belong to the groups N (nervous system) and C (cardiovascular system) in the ATC drug classification system. This is due to the greater risk of causing harm if errors are made with this drugs [34]. Not all of the articles have made it clear what types of therapeutics are involved in errors. The types of drug and the frequency of discrepancy most likely change from ward to ward. Studies suggest that specialised wards, e.g., GI *versus* a general medical ward, tend to be associated with fewer errors, as they usually have a more limited range of illness-specific medicines, and staff at these wards is more experienced in their prescribing [21].

## 4. Conclusions

In the studies reviewed, between 20% and 87% of patients encounter discrepancies at discharge. This shows that there is a problem and one that the healthcare system needs to address. Unquestionably, the number of discrepancies per patient and the types of drugs will differ not only between hospitals, but also between wards at the hospital. A key finding is that as the number of drugs increases, so too does the risk of discrepancies, putting patients with multi-morbid conditions and receiving more than one drug at greater risk. The reasons for medical discrepancies differ, but the most common was related to omission of medication together with discrepancies in route/frequency/dose.
